# Quality of Antipsychotics Prescribing in Dementia Patients in Wakefield (WF9 Area)

**DOI:** 10.1192/bjo.2025.10424

**Published:** 2025-06-20

**Authors:** Bhavana Piparsania, Richard Marriott, Sapan Sunder

**Affiliations:** South West Yorkshire NHS Foundation Trust, Wakefield, United Kingdom

## Abstract

**Aims:** This Quality Improvement Project evaluated antipsychotic prescribing for dementia patients in Wakefield (WF9), comparing local practice with NICE guidelines. NICE recommends antipsychotics for severe agitation/distress, initiated and monitored under specialist supervision for ≥12 months. NHS England data (Jan 2024) shows similar dementia diagnosis rates in Wakefield compared with the national average, but antipsychotic prescribing is ~33% higher regionally. This project investigated this.

Aims were to:

1) Compare antipsychotic prescribing in two WF9 care homes with NICE guidelines (initiation, monitoring, follow-up).

2) Compare local data with NHS England data for Wakefield.

**Methods:** Data from 95 care home residents (Apr 1–Jun 1, 2024) were collected with staff support. Medication charts were reviewed, and data on physical health monitoring and care plans were extracted from System 1. Of 95 residents, 65 were open to community older people’s services, and 30 were not (data from care home records/register). Data were compiled in Excel.

**Results:** Of 95 residents, 66.3% (n=63) had dementia. Among these, 66.4% (n=40) were open to community services. Antipsychotic prescriptions were initiated by local psychiatrists in 58% (n=14/24) of dementia patients; by out-of-area services (unknown) in 21% (n=5/24); and by GPs/hospitals in 21% (n=5/24). Blood monitoring was documented in 56% (n=13/24) and ECG monitoring in 50% (n=12/24). A clear follow-up plan existed for 81% (n=51/63) of dementia patients, but 19% (n=12/63) lacked documented follow-up >1 year. Only 8% (n=2/24) had a documented plan to reduce antipsychotics.

Comparison with NHS England Data: This audit confirmed higher antipsychotic prescribing in Wakefield than the national average. Local data suggested a 4-fold higher rate. ~60% of prescriptions were initiated >1 year prior, and 92% (n=22/24) lacked a clear reduction plan, possibly due to anxiety around reduction (especially by trainees) and limited multidisciplinary input. Regional variations (e.g., Barnsley’s lower rate) raise questions about prescribing thresholds, service differences, and MDT caseload management.

**Conclusion:** Of 95 residents, 66.3% (n=63) had dementia, and 38% (n=24) of those with dementia were on antipsychotics. 8% (n=5/95) were on antipsychotics without a dementia diagnosis. While 81% had follow-up plans and all were monitored for side effects, adherence to NICE guidance for blood/ECG monitoring was suboptimal (56%/50%). Documentation of follow-up and antipsychotic reduction plans requires improvement. A maintained antipsychotic register may improve documentation and ensure appropriate monitoring/review.

